# Global assays of hemostasis in the diagnostics of hypercoagulation and evaluation of thrombosis risk

**DOI:** 10.1186/s12959-015-0038-0

**Published:** 2015-01-23

**Authors:** Elena N Lipets, Fazoil I Ataullakhanov

**Affiliations:** Center for Theoretical Problems of Physicochemical Pharmacology, Russian Academy of Sciences, Moscow, Russia; National Research Center for Hematology, Moscow, Russia; Physics Department, Moscow State University, Moscow, Russia; Federal Research and Clinical Center of Pediatric Hematology, Oncology and Immunology, Moscow, Russia; Faculty of Biological and Medical Physics, Moscow Institute of Physics and Technology, Dolgoprudny, Russia; HemaCore LLC, Moscow, Russia

**Keywords:** Global assays of hemostasis, Hypercoagulation, Thrombosis

## Abstract

Thrombosis is a deadly malfunctioning of the hemostatic system occurring in numerous conditions and states, from surgery and pregnancy to cancer, sepsis and infarction. Despite availability of antithrombotic agents and vast clinical experience justifying their use, thrombosis is still responsible for a lion’s share of mortality and morbidity in the modern world. One of the key reasons behind this is notorious insensitivity of traditional coagulation assays to hypercoagulation and their inability to evaluate thrombotic risks; specific molecular markers are more successful but suffer from numerous disadvantages. A possible solution is proposed by use of global, or integral, assays that aim to mimic and reflect the major physiological aspects of hemostasis process in vitro. Here we review the existing evidence regarding the ability of both established and novel global assays (thrombin generation, thrombelastography, thrombodynamics, flow perfusion chambers) to evaluate thrombotic risk in specific disorders. The biochemical nature of this risk and its detectability by analysis of blood state in principle are also discussed. We conclude that existing global assays have a potential to be an important tool of hypercoagulation diagnostics. However, their lack of standardization currently impedes their application: different assays and different modifications of each assay vary in their sensitivity and specificity for each specific pathology. In addition, it remains to be seen how their sensitivity to hypercoagulation (even when they can reliably detect groups with different risk of thrombosis) can be used for clinical decisions: the risk difference between such groups is statistically significant, but not large.

## Introduction

Thrombotic complications occur in many cases: they accompany or even induce innumerable disorders and states: atherosclerosis, infarction, stroke, pregnancy, trauma, surgery, sepsis, etc. Their danger is presently well recognized, and there are regulations on the use of antithrombotic agents for all patients with thrombotic risks. Such agents are presently available in a huge variety [1] and include direct and indirect coagulation factors inhibitors, antagonists of platelet activation and adhesion receptors, and also of platelet signaling.

However, there are unmet needs with regard to identification the patients at risk of thrombosis, individual selection and correct dosing of these drugs, in particular for individual patients. There is always risk of bleeding (1-3% for all antithrombotics at their recommended doses), and brain hemorrhage can be no less fatal than thrombosis. Traditional coagulation assays are insensitive to hypercoagulation and unable to evaluate thrombotic risks.

A possible solution is proposed by use of global, or integral, assays [2-4] that aim to mimic and reflect the major physiological aspects of hemostasis process in vitro. Here we review the existing evidence regarding the ability of both established and novel global assays (thrombin generation, thrombelastography, thrombodynamics, flow perfusion chambers) to evaluate thrombotic risk in specific disorders. The biochemical nature of this risk and its detectability by analysis of blood state in principle are also discussed.

### Pathophysiology of hypercoagulation and thrombosis

Before speaking about thrombosis risk evaluation and prediction of thrombosis using in vitro diagnostics, it is essential to discuss the biochemical nature of thrombus formation.

#### *Venous thrombosis*

Recent reviews on the venous thrombosis pathogenesis can be found in [5,6]. The basic principles of it were formulated by Virchov in 1859, who defined origin of thrombosis in his famous triad of disorders in blood composition, flow velocity or vascular wall. It is well established that venous thrombi are formed mostly as a result of fibrin formation (so-called ‘red’ thrombi rich in fibrin and entrapped red blood cells) with little if any contribution from platelet adhesion. It is not completely clear, however, how this process is triggered. Venous thrombi are attached to the vessel wall by fibrin [7], and in most cases the wall remains undamaged [8]. The most likely mechanism of triggering thrombosis involves endothelial activation. Upon blood flow stagnation, inflammation and/or hypoxia endothelial cells release the Weibel-Palade bodies containing von Willebrand factor (vWF) of increased length and P-selectin. The release enable attachment of platelets, monocytes, neutrophils [9] and their microparticles. Activated by hypoxia, cytokines, and lipopolysaccharides monocytes express Tissue factor (TF) [10], which stimulates clotting activation. Additional tissue factor is provided by microparticles derived from monocytes, cancer cells [11] and probable neutrophiles [9] depending on the primary disorder. Essential role can be played by contact activation from neutrophil extracellular traps (NET), extracellular chromatin fibers with a backbone of histones [9]; contribution of platelet- and endothelium-derived microparticles is possible [12]. Depending on the balance between procoagulant factors, their inhibitors and fibrinolysis, this endothelium activation might develop into full-scale thrombosis.

#### *Arterial thrombosis*

Arterial thrombosis is usually triggered by rupture of an atherosclerotic plaque. This leads to externalization of collagen, TF and vWF. As a result of rapid flow velocity in arteries, the primary driving mechanism of arterial thrombosis is platelet deposition with fibrin formation playing a secondary, thrombus-stabilizing role [13,14]. This is confirmed by predominance of platelets in such thrombin (so-called ‘white’ thrombi) and by efficiency of antiplatelet agents [15]. Altered platelet adhesion and aggregation due to increased vWF concentration, decreased ADAMTS13 (a metalloproteinase, that cleaves vWF to smaller, less procoagulant forms), increased in vitro platelet aggregation after low concentrations of adenosine diphosphate and/or epinephrine (sticky platelet syndrome) are associated with arterial thrombosis [16,17].

However, there are indications of hypercoagulation state in the plasma of patients suffering from arterial thrombosis: circulating factors XIa and TF were detected in patients following ischemic cerebrovascular events [18], in stable angina patients [19], in patients with systolic heart failure due to ischemic cardiomyopathy [20]. In some of the intravital arterial thrombosis animal models monocyte- and endothelium-derived microparticles were deposited in thrombi [11]. Even double-drug antiplatelet therapy in acute coronary syndrome does not prevent a ~10% yearly risk of a recurrence, while addition of rivaroxaban significantly decreases the risk [21]. These data indicate that blood coagulation contribution in arterial thrombosis should not be neglected.

#### *Microvascular thrombosis*

Originally, pathogenesis of thrombosis was primarily studied in large blood vessels. However, there has been a recent shift in the attention to the microvasculature and to the microcirculatory occlusion [22]. Probably, an important factor in this shift is that the most informative experimental models of thrombosis are microvascular ones [23]. Development of microthrombosis is usually related to TF expression by various cells, degradation of TFPI by neutrophil elastase, fXII activation by NETs released by neutrophils and enhanced by their interaction with platelets [24]. It is observed in multiple diseases (sepsis [25], cancer [26], infarction [27], thrombotic thrombocytopenic purpura [28]) and is a critical factor in the development of disseminated intravascular coagulation and organ dysfunction [29]. Among all types of thrombosis, microvascular thrombosis is most of all associated with plasma ability to coagulate overall increase, hypercoagulability [30].

#### Specific causes of hypercoagulation

Hypercoagulation state, usually meaning increased tendency of blood to clot, can be induced by numerous molecular mechanisms enumerated below.

Hypercoagulation in cancer is usually associated with expression of TF, cancer procoagulant (CP) and adhesion molecules. TF expression is induced by activation of oncogenes or inactivation of tumor suppressor genes [31]. Some of the circulating TF is associated with microparticles [32,33] that also accelerate clotting by providing additional phosphatidylserine. CP is a cysteine protease with factor X activating properties [34]. Its role as a factor predictive of clinical thrombosis has not been successfully demonstrated. Mice models demonstrated that NETs also contribute significantly to hypercoagulation in cancer [35]. Adhesion molecules allow direct interaction of tumor cells with endothelium, platelets, and leukocytes, can induce formation of platelet microthrombi [36].

Normal pregnancy is associated with increase in fibrinogen (Fg), Factors VII, VIII, X, and VWF. Free protein S is decreased secondary to increased levels of its binding protein, the complement component C4b. Plasminogen activator inhibitor type 1 (PAI-1) levels increase 5-fold [37]. Levels of PAI-2, produced by the placenta, increase dramatically during the third trimester [38]. For some complication of pregnancy, increased concentration of endothelial-derived and TF-positive microparticles was reported [39,40].

Hormonal contraceptives increases concentrations of Fg, prothrombin (factor II) and factors VII, VIII and X, and reduction in coagulation inhibitors, such as antithrombin (AT) and protein S and tissue factor pathway inhibitor (TFPI). On the other hand, fibrinolysis is also stimulated: tissue plasminogen activator (tPA) activity is increased, while that of PAI-1 is decreased [41].

Diabetes leads to increase in adhesion and aggregation of platelets, as well as platelet-dependent thrombin formation. Changes in the platelet reactivity occur at the stage of megakariopoiesis. Leukocytes are also activated and expose aminophospholipids and TF, express adhesive molecules that promote interaction of leukocytes with endothelium and formation of leukocyte-platelet aggregates. Dysfunction of endothelium is observed. Concentrations of VWF, factor VII, and Fg are increased, those of AT, PC, endothelial thrombomodulin (TM) are decreased. Platelets, monocytes, endothelium demonstrate increased microparticle production. Levels of PAI-1 and tPA are decreased [42].

To summarize, there are several immediate reasons of high risk systemic thrombosis. First, there are materials that directly activate coagulation: circulating contact pathway-activating microparticles [43], circulating TF on cells or microparticles (in cancer or diabetes), factor XIa (ischemic cerebrovascular events, stable angina), сancer procoagulant, bacteria. The second category includes mechanisms that do not initiate clotting by themselves but may promote clotting propagation by shifting coagulation balance: increased level, activity or longevity of pro-coagulant factors (congenital, pregnancy, oral contraceptives, mutations like prothrombin G20210A [44] or factor V Leiden [45,46]), decreased concentration or function of the anti-coagulant molecules (congenital or consumptive deficiency of AT, PS, PC [47,48]), decreased fibrinolysis, ADAMTS13, increased vWF [16]. The data for several protrombotic states discussed in this article are summarized in Table 1, which attempts to relate mechanism of pro-coagulant changes, disorders that cause them, and the types of thrombosis supported by them.

**Table 1:** **The reasons of hypercoagulation in different states associated with risk of thrombosis discussed in the article**

**Background state**	**Activating material**	**Increased level of procoagulant zymogens**	**Decreased level of coagulation inhibitors**	**Fibrinolysis abnormalities**	**Other hemostasis abnormalities**	**Type of thrombosis**
Cancer	ТF, NET, MP				Сancer procoagulant, adhesive molecules	Venous thrombosis
Pregnancy	TF, МР	Fg, VII, VIII, X	Free protein S	PAI-1↑, PAI-2↑	Thrombocytopenia, platelets activation, VWF↑	Venous thrombosis, Arterial thrombosis
Oral contraceptives		Fg, II, VII, VIII, X	ATIII, PS, TFPI	tPA↑, PAI-1↓		Venous thrombosis
Diabetes mellitus	TF, platelet, monocyte, endothelial МP	Fg, II, V, VII, VIII, and X	ATIII, PC, endothelial ТМ	PAI-1↑, tPA↑	Enhanced platelet adhesion, aggregation, leukocyte activation, VWF↑	Arterial thrombosis, Venous thrombosis?

### Detection of hypercoagulability: possible strategies

Nature of pre-disposition of an individual to thrombosis may be either local or global. Local factors such as vessel wall damage, atherosclerotic plaque formation or blood flow stagnation remain naturally beyond functional laboratory assays of coagulation (although possibility to detect some markers of inflammation and damage in blood indirectly cannot be excluded). Other thrombotic events can be directly associated with global changes in blood composition. These systemic pro-thrombotic changes are called hypercoagulation. When thrombosis can be directly linked to hypercoagulability, several ways to determine it exist.

One way is to detect the specific cause: change in the coagulation factor or coagulation inhibitor concentration, presence of a circulating active factor, microparticles, change in fibrinolysis factor, vWF concentrations. While such research is important, these parameters appear to be innumerable and some of them (e.g. picomolar concentrations of factors) are extremely hard to measure. In addition, isolated information about specific causes does not give an idea about overall tendency of the blood to form thrombi.

Another strategy is to use molecular markers of ongoing thrombosis: D-dimers, fibrinopeptides, soluble fibrin monomers, thrombin-antithrombin complexes or prothrombin fragments. This strategy is used widely and has tremendous clinical advantages, but its major drawback is that it detects traces of coagulation that already occurred or goes presently at full rate. In disseminated intravascular coagulation (DIC), you may have great D-dimer in conjunction with zero clotting due to coagulopathy.

Finally, the natural way to determine hypercoagulation is to carry out coagulation experiments under conditions, where contribution of the hypercoagulation factors is significant (i.e. under conditions close in vivo). It turns out that it is not significant for the traditional clotting assays: when you stimulate clotting with huge concentrations of activators in APTT and PT, contribution of the small quantities of circulating active factors is not essential.

The possible solution to this might be global, or integral, assays of hemostasis [2-4]). They aim to mimic (patho)physiogical processes with greater accuracy and to involve all relevant processes, so they tend to determined overall hemostatic potential. Importantly, these assays usually involve low activator concentration (thrombin generation, TEG) or localization of the activator on the chamber wall (thrombodynamics, flow perfusion chambers). This could indeed make them sensitive to low concentrations of circulating agonists.

The purpose of the present review is to inventory the available data in order to test the existing claim of the global assays on their ability to predict thrombosis.

### APTT and INR: are they indeed inappropriate as global assays?

Till the present times, initial evaluation of hemostatic status is done using APTT and INR assays. They are sensitive, first of all, to deficiencies of coagulation factors that usually result in their prolongation. Shortening of clot times is rare and is often attributed to pre-analytical errors (that play a great role in diagnostics, as it is very easy to induce hypercoagulation by insufficiently careful handling of whole blood). When dealing with thrombosis INR application is usually limited to assess the effectiveness of vitamin K doses [49].

However, there are reports that some thrombotic disorders may be detected by changes in APTT. Mina et al. demonstrated that APTT shortening reliably indicates abnormal factors V, XI, XII, VWF antigen and collagen-binding activity, and the level of procoagulant phospholipids, as assessed using a novel assay procedure (XACT) [50]. It is not clear whether factor VIII increase can shorten APTT. APTT shortening has also been associated with high levels of biochemical markers of thrombin generation and fibrin deposition such as prothrombin fragment 12, thrombin-antithrombin complex, and D-dimer [51]. Tripodi et al showed that shortened APTT is a risk factor for deep vein thrombosis. In patients who had an APTT ratio (coagulation time of test-to-reference plasma) smaller than the fifth percentile of the distribution in controls, the odds ratio (OR) for VTE was 2.4 and was independent of inherited thrombophilic abnormalities. Median APTT ratio values were 0.97 (range: 0.75-1.41) for patients and 1.00 (range: 0.72-1.33) for controls (P < .001) [52]. Prospective observation of a large group (918 patients) with spontaneous venous thrombosis revealed that APTT ratio was significantly longer in patients without thrombosis recurrence (0.97 ± 0.09 vs. 0.93 ± 0.09, P < 0.001) The relative risk (RR) of recurrence among patients with a APTT ratio or < 0.95 was 1.7 [53]. Legnani et al. discovered that venous thrombosis recurrence risk after cancelling anticoagulation was more than twofold higher in patients with ratio < or =0.90 versus those of the reference category (Relative risk (RR): 2.38) [54]. The data indicating predictive value of APTT are given in Table 2.

**Table 2:** **Examples of АPPT ratio response to different procoagulant states**

**Procoagulant state**	**Number of patients in study group**	**Data range in control group, mean ± SD unless otherwise indicated**	**Data range in group with hypercoagulation, mean ± SD unless otherwise indicated**	**Significance**	**Predictive value**	**Reference**	**Comments**
VTE	605 patients, 1290 - controls	Median(range) 1.00(0.72-1.33)	Median(range) 0.97(0.75-1.41)	<0.001	APTT ratio < 0.87 OR = 2.4	[52]	Retrospective study.
Recurrence after first unprovoked VTE	918 with a first VTE 101 – with recurrence	0.97 ± 0.09	0.93 ± 0.09	0.001	APTT ratio < 0.95 RR = 1.79	[53]	Prospective study. Analysis was performed 3 weeks after after completion of anticoagulant therapy.
Recurrence after first unprovoked VTE	628 with a first VTE, 71 – with recurrence				APTT ratio < 0.90 RR = 2.38 compared with APTT ratio > 1.05	[54]	Prospective study. Analysis wasperformed 3-4 weeks after after completion of anticoagulant therapy.
Type 2 diabetes mellitus	60 patients, 57 controls	Median(range) 0.93 (0.71–1.34)	Median(range) 1.03 (0.79–1.27)	0.43		[55]	

An important version of APTT is so-called clot waveform analysis that considers the entire optical density change curve, not only clot time. This assay is even included among the global assays [2-4]. In particular, two-phase curve in this assay is a sensitive and specific predictor of DIC (85% and 92%, respectively) [56]. This curve is explained by precipitation of C-reactive protein with VLDL upon addition of Са [57].

So, it seems that APTT is working in some cases of pro-coagulant changes in plasma, but these successes are mostly associated with changes in the concentrations of coagulation factor components, not with appearance of coagulation-activating circulating material. It is most likely that potent artificial clotting activation in APTT (and even stronger activation in INR) does not allow observation of small effects produced by circulating TF, fXIa, or microparticles. Protein C pathway does not function in APTT unless activated protein C is added, but even then thrombin generation using the same approach is more sensitive [58]. APTT does not include fibrinolysis in any way. Probably that is why APTT has no predictive value as a thrombosis risk marker following surgery [59,60], trauma [61], diabetes [55,62], cancer [63]. Data about pregnancy are contradictive [64,65]. The main hypercoagulation factor detected by APTT shortening is most likely increased concentration or activity of coagulation factor predecessors. For example, in the study [54], increase of the recurrence risk of VT disappeared after adjustment for factor VIII, IX and XI levels, and coagulation factor levels themselves had better predictive capacity of recurrence risk (RR = 2.38 for APTT ratio < 0.90, RR = 3.01; 3.06; 2.14 for increased levels of fVIII, dIX and fXI, respectively). Still, thrombophilic risk factors G1691A-factor V and G20210A-factor II did not differ significantly in groups with normal and shortened APTT [51].

### Hypercoagulation and thrombin generation

Thrombin generation is one of the two best developed and tested global assays of hemostasis. Invented in its present form by the team of Coenraad Hemker of Maastricht University [66], the method uses a thrombin-sensitive chromogenic or, recently, fluorogenic substrate. From the velocity of its cleavage, thrombin concentration as a function of time is obtained and used for diagnostic purposes; it usually has a characteristic bell-shape. Such parameters as endogenous thrombin potential (ETP, area under the thrombin generation curve) are among the most widely used, and their correlation with clinical phenotype is well established. Interestingly, most of the thrombin generation curve is observed after clot formation, and its meaning is still a subject of debate [67].

There are presently numerous modifications of thrombin generation including several commercially available versions. Typically, the assay is performed in platelet free plasma supplemented with phospholipids; use of platelet-rich plasma is also possible. Triggering is done by picomolar TF concentration, although other stimuli can be used. Thrombin generation experiments can be done with TM, protein C-activating enzymes or simply activated protein C to better highlight the protein C pathway.

Tripodi et al. reported that patients with increased thrombin generation in the presence of TM have higher risk of recurrent venous thromboembolism. Those with ETP > 960 nM · min or thrombin peak >193 nM had hazard ratios (HR) for recurrent VTE of 3.41 or 4.57 as compared with those with an ETP <563 nM · min or peak <115 nm. Patients with lag-time <14.5 min had HR of 3.19 as compared with those with lag-time >20.8 min [68]. The same was reported by Besser et al.: after adjustment for D-dimer, thrombophilia, sex, and whether or not the first event was unprovoked, a high ETP remained a significant predictor of recurrence, HR 2.6 [69]. In a similar study of Hron et al., patients without recurrent VTE had lower thrombin generation than patients with recurrence (mean [SD], 349.2 [108.0] nM vs 419.5 [110.5] nM, respectively; P < .001). Compared with patients who had thrombin generation lower than 400 nM, the relative risk (RR) of recurrence was 2.4 [70]. Interestingly, van Hylckama Vlieg et al. did not find any predictive value for thrombosis risk though this might be due to a different experimental design [71]. Chaireti et al. found, paradoxically, that ETP immediately after thrombosis is lower in the group of thrombosis recurrence. If blood was collected 1-2 months after cancellation of anticoagulants, their ETP was insignificantly higher [72].

Increased ETP in platelet-rich plasma was reported for patients after ischemic stroke [73]. In PPP, increased thrombin peak predicted stroke for women and did not correlated with stroke in men (hazard ratio 1.04 for men, 1.7 for women) [74]. ETP is increased in almost any thrombophilia including G20210A mutation [75], AT deficiency [76], factor V Leiden [77] and protein S deficiency [78] (if experiments are with thrombomodulin), upon oral contraceptive use [79], in cancer [80]. ETP is increased in pregnancy [81,82], but seems to reach a plateau in the first trimester [83], while D-dimer, F1 + 2 and TAT increased, and there was no correlation between parameters of ETP assay and markers of in vivo thrombin generation. Lack of this correlation was confirmed in [80]. Patients with diabetes had significantly higher thrombin peak [55,62], probably because of increased level of factors II, V, VII, VIII, and X and decreased protein C [62].

Thrombin peak was reported to correlate with microparticle count, in particular when thrombin generation is performed without adding external activators and phospholipids [55]. Ollivier et al. found that lag time in recalcified plasma is sensitive to TF and does not affect the peak, while peak is sensitive to phospholipids. Distinct contributions of these two factors in cancer patients’ plasmas were differentiated in [84]. Lipopolysaccharides reliably decreased lag time [85].

It appears that thrombin generation is sensitive to various hypercoagulation factors depending on the design: to levels of factors II, V, Fg, AT at high TF (13.6рМ); to fXII, Fg, AT, free TFPI at low TF (1pM) [86], as well as to fVIII and fIX [87]; to protein C pathway defects upon addition of thrombomodulin or protein C activator [88]; to circulating TF when performed without activators; to lipids when performed without externally added lipids. Decrease of activation level increases overall sensitivity but increases deviation. Difference in the mean parameter values for patients with and without thrombosis is usually significant, but SDs usually overlap and it is complicated to transform such result into a clinical recommendation (Table 3). Although thrombin generation test standardization is presently under development [2] its lack restricts the method application.

**Table 3:** **Examples of Trombin generation response to different procoagulant states**

**Procoagulant state**	**Number of patients in study group**	**TG trigger and additional substances**	**Data range in control group, mean ± SD unless otherwise indicated**	**Data range in group with hypercoagulation, mean ± SD unless otherwise indicated**	**Significance**	**Predictive value**	**Reference**	**Comments**
Recurrence after first unprovoked VTE	254 – with a first VTE, 34 - with recurrence	1 pM TF 1 uM PL	ETP, nM∙min 1502 ± 446	ETP, nM∙min 1361 ± 499	0.122	1 tertile compared to the 3 HR = 2.54	[68]	Prospective study. Analysis was performed 2-3 months after completion of anticoagulant therapy.
			IIa max, nM 232 ± 82	IIa max, nM 187 ± 89	0.005	HR = 3.09		
			Tlag, min 12 ± 6	Tlag, min 13 ± 5	0.319	HR = 2.29		
		1 pM TF 1 uM PL 4 nM TM	ETP, nM∙min 986 ± 422	ETP, nM∙min 763 ± 468	0.009	HR = 3.35	[68]	
			IIa max, nM 201 ± 75	IIa max, nM 148 ± 88	<0.001	HR = 4.49		
			Tlag, min 17 ± 7	Tlag, min 19 ± 10	0.174	HR = 2.39		
Unprovoked recurrence after first VTE	188 with a first VTE, 29 – with recurrence	5 pM TF 4 uM PL				ETP > 50th percentile HR = 2.9	[69]	Prospective study. Analysis was performed 2-3 months after completion of anticoagulant therapy.
		5 pM TF 4 uM PL 8nM TM				No significant predictive value	[69]	
Recurrence after first unprovoked VTE	914 with a first VTE, 100 – with recurrence	72 pM TF 3.2 uM PL	IIa max, nM 349 ± 108	IIa max, nM 419 ± 110	<0.001	IIa max >400 nM RR = 2.5	[70]	Prospective study. Analysis was performed after completion of anticoagulant therapy.
First and recurrent VT	187 with a first unprovoked VT 404 controls	1/6 deluted plasma 2.5 pM TF 4 uM PL 1.2 nM TM	Mean ETP(95% CI), nM∙min 1641 (1607 -1676)	Mean ETP(95% CI), nM∙min 1695(1639–1750)		ETP > 90th percentile measured in control subjects DVT HR = 1.7	[71]	Analysis was performed 3 months after completion of anticoagulant therapy.
	173 with a first provoked VT 404 controls		Mean ETP(95% CI), nM∙min 1641 (1607 -1676)	Mean ETP(95% CI), nM∙min 1649(1595-1703)			[71]	
	59 recurrent VTE					HR of recurrence 1.1	[71]	
Recurrence after first unprovoked VTE	105 with a first VTE, 40 – with recurrence	5 pM TF 4 uM PL	ETP, nM∙min 1671 ± 514	ETP, nM∙min 1491 ± 536	0.111		[72]	Prospective study. Analysis was performed upon diagnosis of VTE
			IIa max, nM 302 ± 91	IIa max, nM 261 ± 125	0.058			
			Tlag, min 7.2 ± 2.2	Tlag, min 8.7 ± 5	<0.001			
Acute Ischemic Stroke (men)	42 patients 408 controls	5 pM TF 4 uM PL	geometric mean and interquartile range ETP, nM∙min 1755 (1620 - 1940)	geometric mean and interquartile range ETP, nM∙min 1720 (1572 - 1978)		HR = 0.88/sd	[74]	Prospective study.
			IIa max, nM 327.0 (304.9 - 357.8)	IIa max, nM 330.2 (301.8 - 361.4)		HR = 1.04/sd		
Acute Ischemic Stroke (women)	45 patients 666 controls	5 pM TF 4 uM PL	ETP, nM∙min 1755 (1604 - 1940)	ETP, nM∙min 1863 (1636 -1998)		HR = 1.55/sd	[74]	Prospective study
			IIa max, nM 333.6 (311.0 - 372.4)	IIa max, nM 357.8 (320.5 - 391.5)		HR = 1.71/sd		
Coronary Heart Disease events	186 patients 1000 controls	5 pM TF 4 uM PL	ETP, nM∙min 1765 (1620 - 1940)	ETP, nM∙min 1772 (1604- 1939		HR = 1.09/sd	[74]	Prospective study
			IIa max, nM 333.0 (308.0 - 365.0)	IIa max, nM 330.3 (301.9- 357.8)		HR = 1.02/sd IIa max		
Prothrombin G20210A mutation	148 heterozigote, 111 - controls	6.8 pM TF 30 uM PL	median and interquartile range ETP, nM∙min 1053 (946–1171)	median and interquartile range ETP, nM∙min 1358 (1190–1492)	the carriers as opposed to the non-carriers <0.001		[75]	
			IIa max, nM 292 (267–330)	IIa max, nM 349 (307–385)	<0.001			
			Tlag, min 2.54 (2.46–2.84)	Tlag, min 2.74 (2.46–3.04)	0.268			
	3 homozigote			ETP, nM∙min 1661 (1451–1976)			[75]	
				IIa max, nM 466 (446–470)				
				Tlag, min 3.06 (2.14–5.08)				
AT III-inherited deficiency	9 - controls 18 Type I-IIRS/PE	5 pM TF 4 uM PL	ETP, nM∙min 2200 ± 320	ETP, nM∙min 3366 ± 668	Only Type I-IIRS/PE end controls ETP differs significantlly		[76]	
			IIa max, nM 377.3 ± 49.1	IIa max, nM 493.4 ± 75.0				
	17 -IIHBS heterozygote			ETP, nM∙min 2142 ± 464				
				IIa max, nM 427.2 ± 98.3				
	8 - Cambridge II heterozygote			ETP, nM∙min 2211 ± 268				
				IIa max, nM 391.4 ± 46.8				
VTE in cancer patients	1033 cancer patients 77 VTE cases	71.6 pM TF 3.2 uM PL	median (25th to 75th percentile) ETP, nM∙min 4386 (3804-4890)	median (25th to 75th percentile) ETP, nM∙min 4475 (4087-4915)	0.197	IIa max > 611 nM (75th percentile) HR = 2.1	[80]	Prospective study
			IIa max, nM 499 (360-603)	IIa max, nM 556 (432-677)	0.014			
Type 2 diabetes mellitus	52 patients, 60 controls	1 pM TF 1 uM PL	Median (range) ETP, nM∙min 1844 (1,317–2592)	Median (range) ETP, nM∙min 1835 (1213–2656)	0.96		[55]	
			IIa max, nM 264 (97–432)	IIa max, nM 303 (207–434)	<0.001			
			Tlag, min 7.8 (4.7–18.4)	Tlag, min 5.9 (4.5–11.5)	<0.001			
		1 pM TF 1 uM PL 4 nM TM	ETP, nM∙min 1301 (535–2381)	ETP, nM∙min 1497 (1061–2418)	0.003		[55]	
			IIa max, nM 256 (79–433)	IIa max, nM 297 (216–427)	0.001			
			Tlag, min 10.4 (6.3–25.8)	Tlag, min 7.8 (5.6–13.6)	<0.001			
	43 patients, 60 controls	Ca only	ETP, nM∙min 1678 (539–2231)	ETP, nM∙min 1781 (288–2598)	0.05		[55]	
			IIa max, nM 151 (41–289)	IIa max, nM 202 (128–350)	<0.001			
			Tlag, min 12.6 (7.0–29.5)	Tlag, min 10.8 (7.2–16.1)	<0.001			
Diabetes mellitus	89 patients 49 controls	5 pM TF 4 uM PL	ETP, nM∙min 1566.4 ± 240.7	ETP, nM∙min 1876.5 ± 390.0	<0.001		[62]	
			IIa max, nM 252.8 ± 44.6	IIa max, nM 308.9 ± 39.5	<0.001			
			Tlag, min 4.15 ± 0.74	Tlag, min 3.59 ± 0.62	<0.001			
Normal pregnancy	19 health pregnant women 10 controls	5 pM TF 20 uM PL 0.1 mg/ml CTI	ETP, nM∙min 1553 ± 567	pre-pregnancy ETP, nM∙min 1162 ± 446	Significant difference between pre-pregnancy and early/late pregnancy P < 0.001		[82]	
				IIa max, nM 81 ± 41				
				Early ETP, nM∙min 2157 ± 466				
			IIa max, nM 159 ± 100	IIa max, nM 219 ± 117				
				Late ETP, nM∙min 2410 ± 543				
				IIa max, nM 336 ± 178				
Normal pregnancy	1st Trimester (n = 36)	5 pM TF 4 uM PL	TG on normal pooled plasma was significantly lower than TG on pregnant women. The exact parameter’s values weren’t shown	ETP, nM∙min 2123 ± 335	No significant differences between trimesters		[83]	
				IIa max, nM 366 ± 43				
	2nd Trimester (n = 42)			ETP, nM∙min 2067 ± 326				
				IIa max, nM 374 ± 42				
	3rd Trimester (n = 23)			ETP, nM∙min 1915 ± 261				
				IIa max, nM 336 ± 49				

Fibrinolysis and use of whole blood are currently beyond the available versions of this method, although some preliminary on thrombin generation in whole blood appeared [89]. There are no clinical data for thrombotic states for this version yet.

### Evaluation of thrombosis risk with TEG/ROTEM

The most direct way to characterize clot formation is by rheometry, which has additional advantage of being independent of optical phenomena and easily applied in whole blood. There are numerous rheological approaches, and the best studied of them is thrombelastography. It is the most ancient global assay of hemostasis, where clot formation and platelet aggregation are evaluated simultaneously using forced oscillations rheometry.

Thrombelastography (TEG or ROTEM) has found a wide application for patients undergoing surgery as an alternative to APTT and INR that are not sensitive to hypercoagulation in this state [59,90]. A paper by Yue Dai et al. carefully examines reports between 1980 and 2008 about possibility to predict thrombosis using TEG, and majority of them answered positively. However, sensitivity and specificity varied between 0%-100% and 62%-92% respectively, with odds ratio reported between 1.5 and 27.7 [91] thus preventing meta-analysis. Later reports confirmed predictive value of maximal amplitude (MA) and clot firmness G (G = 5000 MA/100 – MA) as independent indicators of recurrent ischemic stroke after surgery (OR = 1.192, p = 0.022) [92]; the same was obtained for other thrombotic complications Similar data were obtained for ROTEM [90]. MA is believed to be mostly dependent on platelet function and fibrinogen concentration [93]; this might explain why it does not correlate with APTT and INR [92].

TEG revealed hypercoagulation in patients with prostate cancer, in particular in the group with metastases, in agreement with the increase of TF-expressing microparticles. Thrombotic complications arose in 7 out of 22 patients with increased TEG, while APTT and INR were normal [63]. TEG was able to detect hypercoagulation in patients with breast and colorectal cancer [94], gastrointestinal system tumors, respiratory system tumors, and miscellaneous tumors [95], after DVT [96], but not after cerebral venous thrombosis [97]. TEG is increased only in 57% patients with thrombophilia [98], this lack of sensitivity is confirmed in [97,99]. TEG reliably detect hypercoagulation in pregnancy that increases over the whole course [100-102] by parameters r, K, alfa, MA.

Like thrombin generation, thrombelastography is established to detect hypercoagulation, and there is evidence of both its shift in patient groups with known thrombotic risks and in patient groups with clinically occurring thrombosis. The pattern of sensitivity differs from that of thrombin generation: e.g., TEG is better in pregnancy but worse in thrombophilia. Still, wider use of this method suffers from the same shortcomings: deviations between donors are even greater that in thrombin generation resulting in poor difference between risks (Table 4), and there is lack of standardization.

**Table 4:** **Examples of TEG response to different procoagulant states**

**Procoagulant state**	**Number of patients in study group**	**TEG version**	**Data range in control group,** **mean ± SD unless otherwise indicated**	**Data range in group with hypercoagulation, mean ± SD unless otherwise indicated**	**Significance**	**Predictive value**	**Reference**	**Comments**
Patients with acute ischemic stroke	93-Unfavorable outcome evaluatewd by modified Rankin Scale within a year 91-Favorable outcome	Citrate plasma was mixed with kaolin, and loaded in a heparinise-coated cup	means ± SE MA, mm 63.2 ± 0.5	means ± SE MA, mm 66.1 ± 0.6	<0.001	Prediction of unfavorable outcome At higher tertile of MA OR = 1.192	[92]	Prospective study.
Postoperative Thrombotic Complications	240 patients undergoing a wide variety of surgical procedure s, 10 thrombotic complications	celite-activated TEG on native blood samples within 4 min of collection	MA 66 ± 9	MA 71 ± 9			[103]	Prospective study. Thromboelastography was performed immediately after surgery.
	6 myocardial infarction		MA 66 ± 9	MA 74 ± 5		OR = 1.16		
Postoperative Thrombotic Complications	152 critically ill patients in the surgical intensive care unit 16 thrombotic complications	native blood, rTEG(activation with kaolin, human recombinant TF, phospholipids)				G > 12.4 dynes/cm OR = 1.25	[104]	
Normal pregnancy	65/65	Recalcified citrate plasma	R, min 7.8 ± 2.5	R, min 6.1 ± 1.8	<0.001		[102]	
			K, min 2.7 ± 2.3	K, min 1.4 ± 0.5				
			Alfa, deg 57.7 ± 11.6	Alfa, deg 70.6 ± 6.5				
			MA, mm 61 ± 5.9	MA, mm 71 ± 3.8				
			Ly 30, % 0.8 ± 1.7	Ly 30, % 0.3 ± 0.7				

### Novel assays

There are several innovative global assays that are not used widely yet but may be promising as they include some really important aspects. Some of them are versions of the existing ones (e.g. there are numerous rheometric approaches beyond TEG [105]), while others use completely innovative principles. Below we discuss the methods that have been tested with regard to the pro-thrombotic sensitivity.

#### Thrombin-and-plasmin generation

There are several versions of the method to detect thrombin and plasmin at once [106-108]. Increased coagulation and impaired fibrinolysis are detected by means of overall hemostasis potential in patients with acquired arterial thrombotic events and vasculopathies, such as diabetic patients with microvascular complications, 15 patients with preeclampsia, 16 and elderly female patients with coronary heart disease [106]. Although the data are very scarce, this method is interesting as the only known alternative to TEG with regard to fibrinolysis evaluation.

#### *Thrombodynamics*

A novel strategy of blood coagulation testing is proposed in the thrombodynamics assay that has been developed and used as a research tool for almost two decades and became commercially available for clinical labs in 2012. The central idea of the method is monitoring spatial fibrin formation initiated by immobilized tissue factor in plasma by videomicroscopy [109], so that clot is initially formed on the activator and then propagates into plasma. A version of the assay exists that can determine thrombin formation as a function of time and space in parallel with fibrin [110].

The idea behind this is to take into account spatial heterogeneity of blood coagulation, in other words, the fact that clotting initiation and propagation occurs in spatially separated regions [111]. In agreement with the wound clotting in vivo, tissue factor is located on the surface, and clot propagates because of coagulation factor activation and diffusion [112]. Importantly, separation of the activation and propagation phases makes the assay particularly sensitive to the presence of coagulation activators in plasma such as circulating tissue factor [112] or factor XIa [43]. Spatial clot formation velocity indicates overall procoagulant potential, while formation of activator-independent spontaneous clotting centers may indicate presence of microparticles and long-lived coagulation factors [43]. Pre-analytical standardization for this assay has also recently become available [113].

These biochemical findings have been confirmed by several preliminary studies. Hypercoagulation detected by thrombodynamics in patients with sepsis was confirmed by subsequent increase of D-dimers and occasional thrombosis [114]. Spontaneous clotting and increase spatial clot growth velocity were observed in patients with well-known thrombotic risks suffering from lymphomas, lymphogranulomatosis, thrombophilia, hemolytic anemia, acute leukemia, cardiac infarction [43]; the same was observed in a detailed study of multiple myeloma [115]. A case study demonstrated ability of thrombodynamics to detect hypercoagulation in beta-thalassemia [116]; portal vein thrombosis occurred after several weeks of increased clot growth velocity. In some of these papers, comparison was done with thrombin generation and thrombelastography, which did not show hypercoagulation changes in most cases.

To summarize, thrombodynamics assay shows significant promise as a tool to detect hypercoagulation and evaluate thrombotic risk, but additional clinical research is needed to establish a reliable relationship between assays indicators and thrombosis risks.

#### Flow perfusion chambers

Formation of platelets-and-fibrin thrombi in flow chambers observed by microscopy is potentially an “ultimate” global assay that is able to evaluate both platelet functions (including adhesion, aggregation, and procoagulant activity) and blood coagulation. Such microfluidic devices are being actively developed and used for various applications (see recent review in [116]). Review of this rapidly developing field is beyond the scope of the present paper. However, it should be noted that there are reports on the ability of flow perfusion chambers to detect hypercoagulation changes in blood [117-119]. However, there are few clinical studies and standardization status of these chambers is very poor [120]. Although theroretical consideration suggest significant potential of flow chambers, they have a long way to go in order to become a clinical tool.

## Conclusions

The first conclusion of the present analysis is that the claim of the global assays that they can detect functional hypercoagulation is valid, to a significant degree. Compared with INR and APTT, sensitivity of the new global assays to hypercoagulation is definitely higher and encompasses a wider range of disorders and hypercoagulation causes. It is likely that there are two reasons behind this. First, these novel assays use smaller concentrations of activators, which do not obscure the effect of circulating pro-coagulant material (or, in some cases, activation and propagation phases are spatially separated). Second, the parameters provided by new global assays might be more sensitive than just time of clot formation, and involvement of all major reactions makes the assays sensitive to other pro-coagulant changes.

However, there are two major issues that complicate use of global assays for the evaluation of thrombotic risks.

The most important concern is that conclusion about sensitivity of the assays is usually reached with large patient cohorts only, standard deviations are large, and the differences of mean assay parameters between groups are significant mostly because of large statistics (Tables 2, 3 and 4). In other words, if we attempt to define boundaries and select patient groups with different risks based on the assay parameters, the overall difference of thrombotic risk between groups is not usually large. It is questionable whether this difference is sufficient to affect clinical decisions. Some of the novel assays show promise of sensitivity increase, but their clinical utility also remains to be tested directly.

Another concern is lack of standardization. There are numerous versions for each assay, and clinical investigations often use different approaches, and the assay sensitivity strongly depends on the protocol used. Therefore, the results from different papers cited in this review might be difficult to interpret and to reproduce. The current attempts at standardization for some of the better established global assays such as thrombin generation [121-123]) make us hope that this might be resolved in the foreseeable future.

## Abbreviations

APTT: Activated partial thromboplastin time
AT: Antithrombin
CP: Cancer procoagulant
DIC: Disseminated intravascular coagulation
DVT: Deep venous thrombosis
ETP: Endogenous thrombin potential
F1 + 2: Prothrombin activation fragments
HR: Hazard ratio
INR: International normalized ratio
MP: Microparticles
NET: Neutrophil extracellular traps
PAI-1: Plasminogen activator inhibitor type 1
PC: Protein C
PPP: Platelet poor plasma
PS: Protein S
PT: Prothrombin time
ROTEM: Rotational thromboelastometry
RR: Relative risk
TAT: Thrombin-antithrombin complex
TEG: Thromboelastography
TF: Tissue factor
TFPI: Tissue factor pathway inhibitor
TM: Thrombomodulin
tPA: Tissue plasminogen activator
VLDL: Very low density lipoproteins
VT: Venous thrombosis
VTE: Venous thromboembolism
vWF: Von Willebrand factor
